# The Pentapeptide RM-131 Promotes Food Intake and Adiposity in Wildtype Mice but Not in Mice Lacking the Ghrelin Receptor

**DOI:** 10.3389/fnut.2014.00031

**Published:** 2015-01-12

**Authors:** Katrin Fischer, Brian Finan, Christoffer Clemmensen, Lex H. T. van der Ploeg, Matthias H. Tschöp, Timo D. Müller

**Affiliations:** ^1^Institute for Diabetes and Obesity (IDO) and Helmholtz Diabetes Center, Helmholtz Center Munich, German Research Center for Environmental Health (GmbH), Neuherberg, Germany; ^2^Division of Metabolic Diseases, Department of Medicine, Technische Universität München, Munich, Germany; ^3^Rhythm Pharmaceuticals Inc., Boston, MA, USA

**Keywords:** ghrelin, RM-131, food intake, adiposity, GHSR1a, GHR

## Abstract

The gastrointestinal peptide hormone ghrelin is the endogenous ligand of the growth hormone secretagogue receptor (a.k.a. ghrelin receptor, GHR). Currently, ghrelin is the only circulating peripheral hormone with the ability to promote a positive energy balance by stimulating food intake while decreasing energy expenditure and body fat utilization, as defined in rodents. Based on these and additional, beneficial effects on metabolism, the endogenous ghrelin system is considered an attractive target to treat diverse pathological conditions including those associated with eating/wasting disorders and cachexia. As the pharmacological potential of ghrelin is hampered by its relatively short half-life, ghrelin analogs with enhanced pharmacokinetics offer the potential to sustainably improve metabolism. One of these ghrelin analogs is the pentapeptide RM-131, which promotes food intake and adiposity with higher potency as compared to native ghrelin in rodents. Whereas, the effect of RM-131 on energy metabolism is solidly confirmed in rodents, it remains elusive whether RM-131 exerts its effect solely via the ghrelin receptor. Accordingly, we assessed the receptor specificity of RM-131 to promote food intake and adiposity in mice lacking the GHR. Our data show that in wildtype mice RM-131 potently promotes weight gain and adiposity through stimulation of food intake. However, RM-131 fails to affect food intake and body weight in mice lacking the GHR, underlining that the anabolic effects of RM-131 are mediated via the ghrelin receptor in mice.

## Introduction

The gastrointestinal peptide hormone ghrelin is a key peripheral hormone implicated in a myriad of metabolic functions. Presently, ghrelin is the only known peripheral hormone that promotes body weight gain and adiposity through stimulation of food intake while decreasing energy expenditure and lipid utilization (1). In addition to its ability to regulate energy metabolism via hypothalamic (2–4) and non-hypothalamic (5, 6) neurocircuits, ghrelin exerts a series of effects on metabolism. Accordingly, ghrelin acutely promotes the release of growth hormone from the anterior pituitary (7) regulates glucose metabolism upon chronic systemic treatment (8, 9), stimulates lipogenesis in white adipose tissue (10), acutely enhances gastric acid secretion and gut motility (11–13), modulates reward seeking behavior and taste sensation (14–17), lowers non-shivering thermogenesis of brown adipose tissue (18, 19), has cardioprotective effects (20–22), and protects against muscle atrophy (23, 24). In line with a growing number of preclinical and clinical studies, the numerous beneficial effects of ghrelin make the endogenous ghrelin system an attractive therapeutic target for a series of pathological conditions. As such, ghrelin and its analogs might offer potential to treat cachexia (25, 26), sarcopenia (27), myopenia (28), gastroparesis (29), or anorexia nervosa (26) whereas inhibition of ghrelin signaling might offer potential to treat obesity and diabetes (30).

Ghrelin promotes its biologic action via activation of the growth hormone secretagogue receptor (GHSR, also referred to as the ghrelin receptor; GHR), a seven transmembrane G protein-coupled receptor with wide distribution in human tissues (31, 32). A spliced variant of the GHR, the GHSR1a seems to be the only endogenous active ghrelin receptor, since neither central (33) nor peripheral (34) ghrelin administration affects systems metabolism in mice lacking the GHR gene. To bind and activate its receptor, ghrelin requires acylation of its serine 3 residue with an n-octanoic or n-decanoic acid (7), a post-translational modification achieved by the ghrelin-O-acyltransferase (GOAT) (35, 36). Whereas, the pharmacological potential of GHR pathway modulation has been emphasized in a series of preclinical and clinical studies [as reviewed in Ref. (26, 37)], the efficacy of acyl-ghrelin to improve systems metabolism is hampered by its relatively short half-life, which, depending on the species, varies between 30 min in rats and 240 min in humans (38).

Whereas, the pharmacological potential of acyl-ghrelin is limited by its rapid degradation and the fragility of its serine 3 acylation, ghrelin mimetics offer potential to more sustainably improve systems metabolism. The pentapeptide RM-131 (BIM-28131) confers increased bioavailability and improved pharmacokinetic properties as compared to the native peptide. RM-131 has a high *in vitro* affinity to bind and activate GHSR1a (39) and its chronic systemic administration in rodents increases body weight gain and adiposity through stimulation of food intake (40–42). The orexigenic effects of RM-131 are mediated by stimulation of meal size, meal numbers, and meal duration (41, 43) and are paralleled by increased c-fos immunoreactivity in arcuate nucleus AgRP/NPY neurons (43). Similar to ghrelin, RM-131 stimulates growth hormone release from the anterior pituitary (43), increases GI motility and gastric emptying (39, 44, 45), and reduces inflammation and tissue wasting in animal models of cachexia (40, 46, 47) and of inflammatory bowel disease (39, 40). Compared to native human ghrelin, RM-131 is about 10- to 100-fold more potent, and it is 600- to 1,800-fold more potent compared to ghrelin mimetics tested thus far in clinical trials (39). The first clinical studies using RM-131, which is in phase 2 clinical trials for the treatment of diabetic gastroparesis and intestinal dysmotility disorders, report that a single subcutaneous administration of RM-131 accelerates gastric emptying in type 2 diabetic patients with gastroparesis (45). These and a series of preclinical data support the therapeutic potential of RM-131 to improve systems metabolism. However, uncertainty remains about whether RM-131 exerts its *in vivo* effects solely via GHSR1a signaling. In this study, we assessed whether the anabolic action of RM-131 is exclusively attributable to GHSR1a signaling in mice. In line with previous reports, we show that RM-131 potently enhances both acute and chronic food intake and adiposity in wildtype (wt) mice. We show that RM-131 has no effect on food intake, body weight, and adiposity in mice lacking the GHR. In summary, our data support the therapeutic potential of RM-131 to accelerate caloric intake and weight gain and indicate that the *in vivo* anabolic effects of RM-131 are exclusively mediated via the ghrelin receptor in mice.

## Materials and Methods

### Animals and acute food intake assessments

All animal experiments and procedures were approved by the Animal Use and Care Committee of Bavaria, Germany. The mice were on a pure C57BL/6J background and were bred in house under standard laboratory conditions (constant humidity, 12/12 h light dark cycle, 22 ± 1°C). For the measurement of 24 h acute food intake, non-fasted mice were single housed whereas for the 7-day chronic study the mice were double-housed. All test compounds were solved in H_2_O containing 5% mannitol. The effect of RM-131 on acute food intake was assessed in 10-week-old male chow-fed C57BL6/J wt (*N* = 40; 26.71 ± 0.14 g) and GHR knock-out (ko) mice (*N* = 40; 26.75 ± 0.22 g). Mice were matched for body weight and body fat mass (*N* = 8 each group) and treated with a single subcutaneous injection of either rat ghrelin (500 or 5,000 nmol/kg, PolyPeptide Laboratories, Strasbourg, France), RM-131 (250 or 500 nmol/kg) or vehicle control (H_2_O containing 5% mannitol). Acute food intake was measured at time points 0.5, 1, 2, 4, 8, 12, and 24 h post-ghrelin treatment.

### Chronic peripheral treatment of RM-131 in wt and GHR ko mice

The chronic effect of RM-131 on body weight, body composition (fat and lean mass), and food intake was measured in 13-week old male chow-fed C57BL6/J wt (*N* = 32; 28.09 ± 0.2 g) and GHR ko mice (*N* = 32; 28.36 ± 0.24 g). The mice were matched for body weight and body fat mass (*N* = 8 each group) and treated for seven consecutive days via daily subcutaneous injections of either rat ghrelin (5,000 nmol/kg), RM-131 (50 or 500 nmol/kg), or vehicle (H_2_O containing 5% mannitol). Body composition (fat and lean tissue mass) was assessed at study day 7 using MRI technology (EchoMRI, Houston, TX, USA) as previously described (48).

### Data analysis

Differences between treatment groups were assessed by one-way or two-way ANOVA followed by Bonferroni *post hoc* test as appropriate. All results are given as means ± SEM. Results were considered statistically significant when *p* < 0.05, with the significance level indicated as *(*p* < 0.05), **(*p* < 0.01), and ***(*p* < 0.001).

## Results

### RM-131 stimulates acute food intake in wt but not GHR ko mice

**Figure 1:**
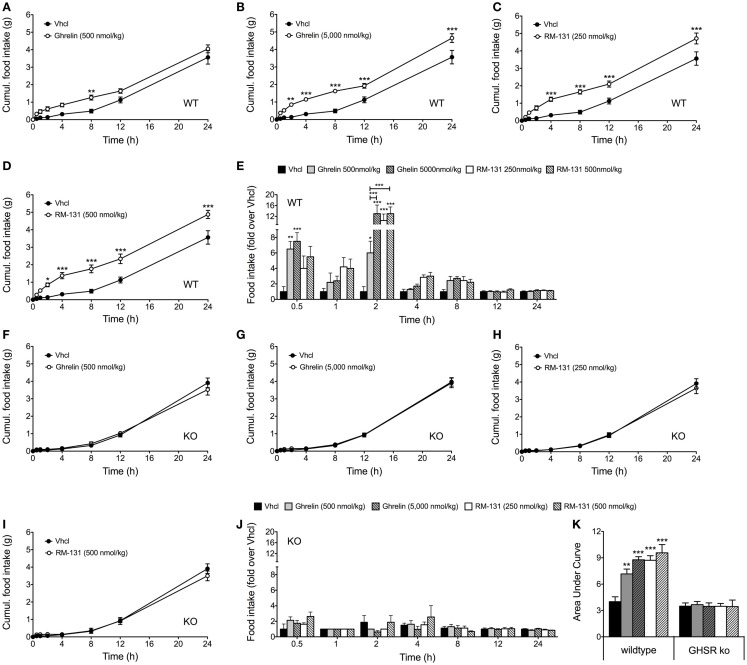
**Acute effect of RM-131 on food intake in wt and GHR ko mice**. Acute 24 h food intake in C57Bl/6J wt and GHR ko mice treated with a single subcutaneous injection of vehicle, ghrelin (500 or 5,000 nmol/kg) or RM-131 (250 or 500 nmol/kg) **(A–K)**. Effects on 24 h cumulative food intake **(A–D,F–I)**, fold-increase in food intake relative to mice treated with vehicle **(E,J)**, and area under curve of the 24-h food intake **(K)**. *N* = 8 mice each genotype and treatment group. Data represent means ± SEM. Asterisks indicate *p  < 0.05; **p < 0.01; ***p < 0.001.

To assess the effects of RM-131 and ghrelin on acute food intake, C57Bl/6J wt and GHR ko mice were treated with a single subcutaneous injection of either rat ghrelin (500 and 5,000 nmol/kg), RM-131 (250 and 500 nmol/kg), or vehicle control. In wt mice, we observed an immediate dose-dependent increase in acute food intake following administration of both ghrelin (Figures 1A,B) and RM-131 (Figures 1C,D). For both ghrelin and RM-131, the greatest peak of compound-stimulated food intake was observed 2 h post-injection (Figure 1E). In line with previous reports indicating that RM-131 has a greater potency to promote food intake relative to native ghrelin (42), we observed a greater increase in food intake in mice treated with 500 nmol/kg RM-131 (area under curve *p* < 0.001, Figure 1K) as compared to an equimolar dose of native ghrelin (area under curve *p* < 0.01, Figure 1K). Importantly, we observed neither for RM-131 nor for ghrelin an effect on acute food intake in the GHR ko mice (Figures 1F–K) with a similar 24 h time-course of food intake between the vehicle treated wt mice and all treatment groups of the GHR ko mice (all *p* > 0.05).

### Chronic systemic administration of RM-131 promotes body weight gain and adiposity through GHR signaling

**Figure 2:**
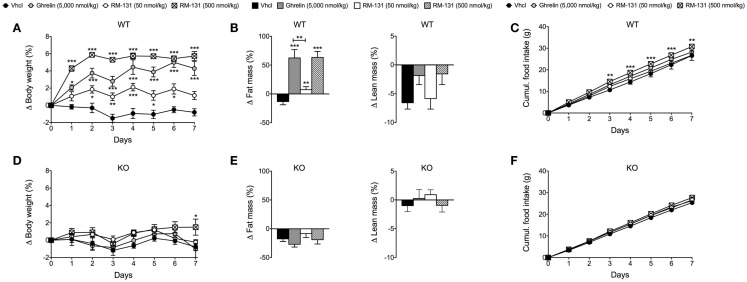
**Chronic effect of RM-131 on food intake, body weight, and body composition in C57BL/6J wt and GHR ko mice**. Body weight, body composition (fat and lean tissue mass), and cumulative food intake in wt **(A–C)** and GHR ko mice **(D–F)** treated with daily subcutaneous injections of ghrelin (5,000 nmol/kg), RM-131 (50 or 500 nmol/kg), or vehicle control. *N* = 8 each genotype and treatment group. Data represent means ± SEM. Asterisks indicate *p  < 0.05; **p < 0.01; ***p < 0.001.

To assess the chronic effects of RM-131 on food intake and adiposity, C57Bl/6J wt and GHR ko mice were treated for seven consecutive days with once daily subcutaneous injections of RM-131 (50 and 500 nmol/kg), rat ghrelin (5,000 nmol/kg), or vehicle control. In wt mice, administration of rat ghrelin (5,000 nmol/kg) significantly increased body weight and body fat mass relative to vehicle treated controls (Figures 2A,B). Neither food intake nor lean tissue mass was different between the ghrelin treated wt mice and vehicle treated controls (Figures 2B,C). Treatment of wt mice with RM-131 at concentrations of 50 or 500 nmol/kg both increased body weight relative to vehicle controls (both *p* < 0.01). However, body weight gain was significantly greater in mice treated with 500 nmol/kg RM-131 compared to mice treated with 50 nmol/kg RM-131 (*p* < 0.0001) and as compared to mice treated with a 10-fold higher dose of ghrelin (5,000 nmol/kg; *p* < 0.05) (Figure 2A). As in the ghrelin treated mice, mice treated with RM-131 displayed an increase in body weight that was a result of an increase in body fat but not lean tissue mass (Figure 2B). Notably, food intake was increased only in wt mice treated with 500 nmol/kg RM-131 (*p* < 0.05) but not in mice treated with 50 nmol/kg RM-131 or 5,000 nmol/kg ghrelin (Figure 2C). In line with our study showing that RM-131 promotes food intake exclusively via the GHR, we observed no effect of RM-131 at concentrations of 50 or 500 nmol/kg on body weight (Figure 2D), body composition (Figure 2E), or food intake (Figure 2F) in the GHR ko mice relative to their vehicle treated controls.

## Discussion

A series of preclinical and clinical data support the therapeutic potential of ghrelin and its analogs to improve pathological conditions associated with eating/wasting disorders and cachexia (26), intestinal dysmotility disorders (49), and diabetic gastroparesis (39, 44, 45). However, whereas the therapeutic potential of GHSR1a pathway modulation is generally acknowledged, the pharmacological value of native ghrelin is limited by its rapid degradation and the fragility of its serine 3 acylation. Several clinical studies report beneficial effects of ghrelin administration on food intake in healthy individuals (50–52), patients with anorexia nervosa (53), and in patients with cachexia associated with cancer (54), chronic obstructive pulmonary disease (COPD) (55) and renal failure (56, 57). However, not all studies were able to replicate these findings (25, 58, 59). Potential pitfalls of these studies are the limited amount of patients analyzed and the overall short duration of ghrelin treatment. Nevertheless, based on the relative short half-life of the native peptide, ghrelin mimetics, which overcome these issues are predicted to more sustainably improve metabolic and other clinical parameters. Several clinical studies have assessed the therapeutic potential of ghrelin mimetics, such as anamorelin (RC-1291) (52), ulimorelin (TZP-101) (60–62), and RM-131 (44, 45) for the treatment of cachexia, gastroparesis, and gastric/intestinal dysmobility disorders. Whereas, TZP-101 had only limited success to improve gastroparesis in a large-scale clinical study (63), the pentapeptide RM-131, exerts superior effects on systems metabolism as compared to native ghrelin and other ghrelin mimetics so far tested in clinical trials (39). However, it remains elusive whether RM-131 exerts its *in vivo* anabolic effects exclusively via GHR activation. Accordingly, the aim of this study was to assess whether RM-131 promotes food intake and adiposity via GHR signaling *in vivo*. In line with previous studies, our data show that in wt mice, RM-131 potently enhances food intake, body weight, and adiposity. We show that RM-131 has no effect on food intake and body weight gain in mice lacking the GHR (including the active/spliced variant GHSR1a), indicating that the anabolic effects of RM-131 are exclusively mediated via the ghrelin receptor in mice.

In summary, our data align with numerous reports indicating that GHSR1a pathway modulation is a promising and powerful tool to promote food intake and adiposity, underscoring the therapeutic potential of manipulating the endogenous ghrelin system to treat pathological conditions associated with excessive tissue wasting and cachexia. Our data also support previous findings indicating that RM-131 promotes its biological action at concentrations 10- to 100-fold lower than native ghrelin. However, whereas our studies clearly show that RM-131 promotes food intake and adiposity exclusively via the ghrelin receptor in mice, more studies are warranted to assess whether more complex functions of ghrelin, such as the regulation of reward seeking behavior, taste sensation, and protection of muscle atrophy, are mediated via RM-131-GHSR1a interaction. In addition, based on the superior anabolic efficacy of RM-131 compared to native ghrelin, RM-131 holds promise for proof of concept experiments in mice to evaluate potentially clinically beneficial effects of RM-131 in diet-induced obesity, with relative ghrelin resistance (64).

## Author Contributions

Katrin Fischer, Brian Finan, Christoffer Clemmensen, and Timo D. Müller conducted the experiments, participated in study design and interpretation of the data, and drafted the manuscript. Matthias H. Tschöp and Lex H. T. Van Der Ploeg participated in conceiving the experiments, revised the data critically, and helped editing the manuscript.

## Conflict of Interest Statement

Lex H. T. Van der Ploeg is an employee of Rhythm Pharmaceuticals, Inc., which is developing RM-131 for the treatment of diabetic gastroparesis and other functional gastrointestinal disorders in humans. The other authors have no conflicts of interest to disclose.
